# Resurgence of malaria infection after mass treatment: a simulation study

**DOI:** 10.1186/s12936-019-3019-0

**Published:** 2019-12-05

**Authors:** Thomas A. Smith, Peter Pemberton-Ross, Melissa A. Penny, Nakul Chitnis

**Affiliations:** 1 Department of Epidemiology and Public Health, Swiss Tropical and Public Health Institute, 4051 Basel, Switzerland; 2 University of Basel, Petersplatz 1, Basel, Switzerland; 3 Present Address: Amgen Europe GmbH: Rotkreuz, Zug, Switzerland

**Keywords:** *Plasmodium falciparum*, Simulation, Elimination

## Abstract

**Background:**

Field studies are evaluating if mass drug administration (MDA) might shorten the time to elimination of *Plasmodium falciparum* malaria, when vector control measures and reactive surveillance strategies are scaled-up. A concern with this strategy is that there may be resurgence of transmission following MDA.

**Methods:**

A conceptual model was developed to classify possible outcomes of an initial period of MDA, followed by continuously implementing other interventions. The classification considered whether elimination or a new endemic stable state is achieved, and whether changes are rapid, transient, or gradual. These categories were informed by stability analyses of simple models of vector control, case management, and test-and-treat interventions. Individual-based stochastic models of malaria transmission (*OpenMalaria*) were then used to estimate the probability and likely rates of resurgence in realistic settings. Effects of concurrent interventions, including routine case management and test-and-treat strategies were investigated.

**Results:**

Analysis of the conceptual models suggest resurgence will occur after MDA unless transmission potential is very low, or the post-MDA prevalence falls below a threshold, which depends on both transmission potential and on the induction of bistability. Importation rates are important only when this threshold is very low. In most *OpenMalaria* simulations the approximately stable state achieved at the end of the simulations was independent of inclusion of MDA and the final state was unaffected by importation of infections at plausible rates. Elimination occurred only with high effective coverage of case management, low initial prevalence, and high intensity test-and-treat. High coverage of case management but not by test-and-treat induced bistability. Where resurgence occurred, its rate depended mainly on transmission potential (not treatment rates).

**Conclusions:**

A short burst of high impact MDA is likely to be followed by resurgence. To avert resurgence, concomitant interventions need either to substantially reduce average transmission potential or to be differentially effective in averting or clearing infections at low prevalence. Case management at high effective coverage has this differential effect, and should suffice to avert resurgence caused by imported cases at plausible rates of importation. Once resurgence occurs, its rate depends mainly on transmission potential, not on treatment strategies.

## Background

**Fig. 1:**
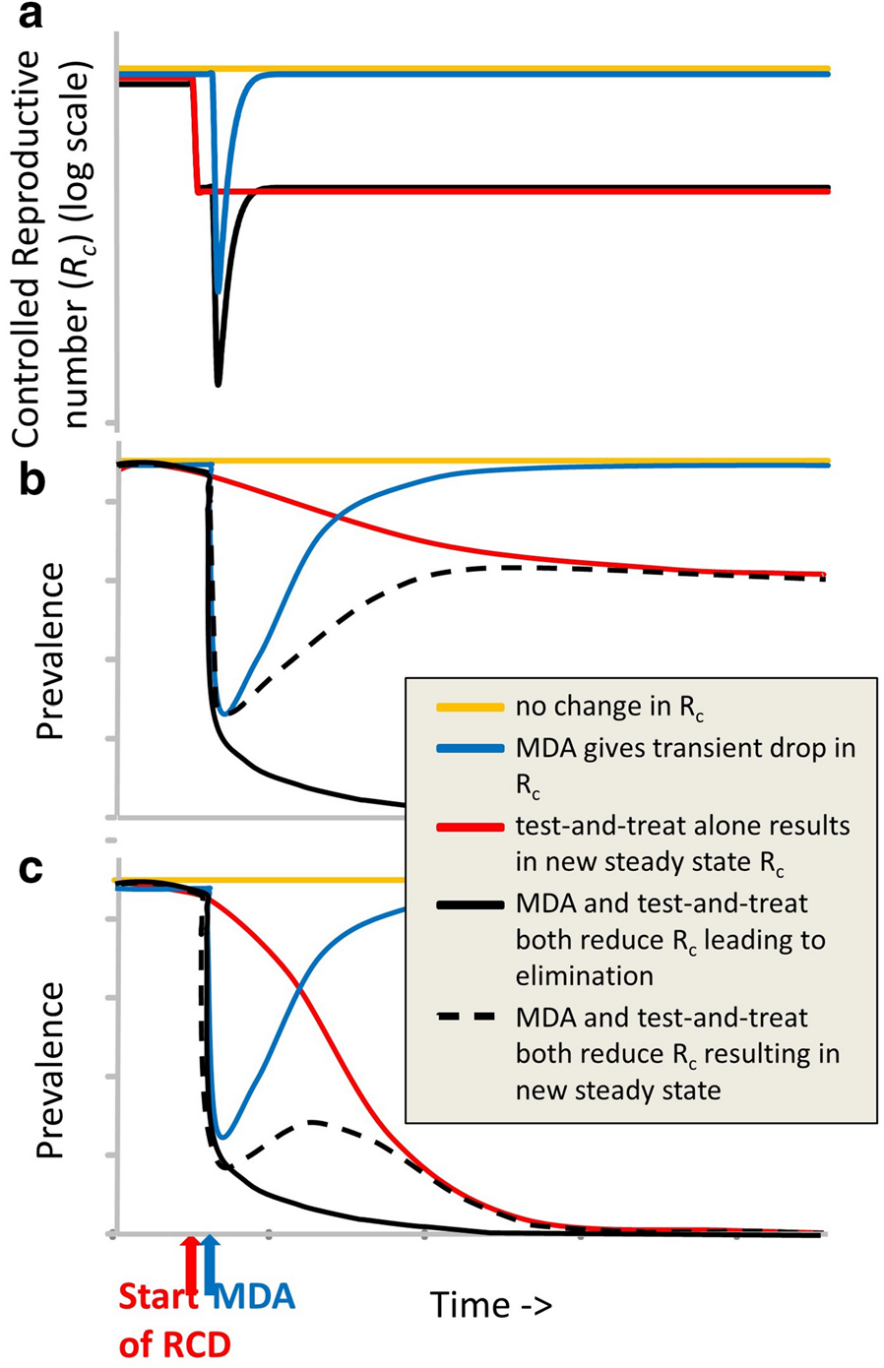
Hypothetical time profiles of *R*_*c*_ and prevalence resulting from MDA and test-and-treat strategies. **a** Time course of average transmission potential represented by the controlled reproduction number, $R_{c}$. **b** Time course of prevalence where test-and-treat alone does not result in elimination. **c** Time course of prevalence where test-and-treat alone may eventually result in elimination. Colours represent different scenarios

Substantial reductions in *Plasmodium falciparum* malaria transmission in Africa have been achieved by rolling out insecticide-treated nets and scaling up access to artemisinin-based combination therapy [1]. In some previously highly endemic areas where transmission levels have been substantially reduced, additional intervention strategies are being trialled with a view to achieving elimination. In particular, in Southern Province of Zambia, the application of 3 dry-season rounds of mass test-and-treat with artemether–lumefantrine was trialled, but failed to interrupt transmission [2]. A recent trial aims to test if the achievement of elimination by such a strategy can be accelerated by supplementing it with mass drug administration (MDA) using a longer lasting anti-malarial combination (dihydroartemisinin–piperaquine, DHA-P) [3, 4]. In an otherwise stable environment, the maximal impact on transmission of a malaria intervention programme generally occurs shortly after maximal scale-up [5] (Fig. 1).

This supports front-loading of drug-based elimination programmes and is the reason why malaria elimination programmes in the mid-20th century envisaged an intense initial phase that would break transmission [6, 7]. Consequently, the MDA under investigation is conceived of as a time-limited pulsed intervention, with the objective of shortening timelines, primarily to accelerate the achievement of elimination. Though there have been trials of long-term preventive chemotherapy in endemic settings [8], MDA is not feasible as a long-term recurrent intervention, since it is difficult to organize and obtain consent for recurrent treatment of uninfected people at high coverage; also, it is expensive and potentially selects drug-resistant parasites [9, 10]. Even if transmission is interrupted by the MDA, in the absence of effective surveillance reintroductions will occur and both *R*_*c*_ and prevalence will return to the initial stable states (blue lines in Fig. 1a–c) [5, 9]. This phenomenon is referred to as resurgence [11, 12].

Many different strategies might be considered as ways of averting resurgence. Most of these can be classified as either (i) vector control approaches, that reduce transmission potential without directly impacting the reservoir of infection in the human population; or (ii) chemoprevention strategies that provide both prophylaxis and clear new infections that have arisen. The latter might include recurrent focal MDA, focal test-and-treat, or reactive case detection (RCD) strategies, in which neighbours of passively detected cases are either presumptively treated or screened for parasites and treated if positive.

This paper reports a conceptual model developed for classifying the possible outcomes when an enhanced intervention strategy (consisting of either intensified vector control, or chemoprevention) is initiated alongside a pulse of one or more rounds of high-coverage MDA. The conceptual model draws on the results of stability analyses that have been carried out for a range of malaria models. A large-scale simulation experiment using the *OpenMalaria* (https://github.com/SwissTPH/OpenMalaria/wiki) microsimulation platform was then used to determine which of these outcomes are likely in real-world settings. The parameterizations were based on data from Southern Zambia [4, 13, 14]. In particular, simulated time-series’ of prevalence were analysed to identify which characteristics of interventions determine whether their effectiveness in averting or slowing down resurgence.

## Methods

### Conceptual model

The conceptual model was illustrated by graphical representations of possible temporal trends in malariological indices induced by combining initial MDA with additional continual or recurrent interventions (Fig. 1). For simplicity, this figure neglects seasonality. For conciseness, the package of additional interventions, (which in principle may comprise vector control and/or chemoprevention programmes), is referred to as test-and-treat. The indices considered were (i) transmission potential, *R*_*c*_, defined as the average number of secondary infections due to each primary infection in the presence of control measures [15], and equal to the basic reproduction number, *R*_0_, reduced proportionately by the effect of the interventions on each of the parameters; and (ii) the corresponding prevalence of infection.

**Fig. 2:**
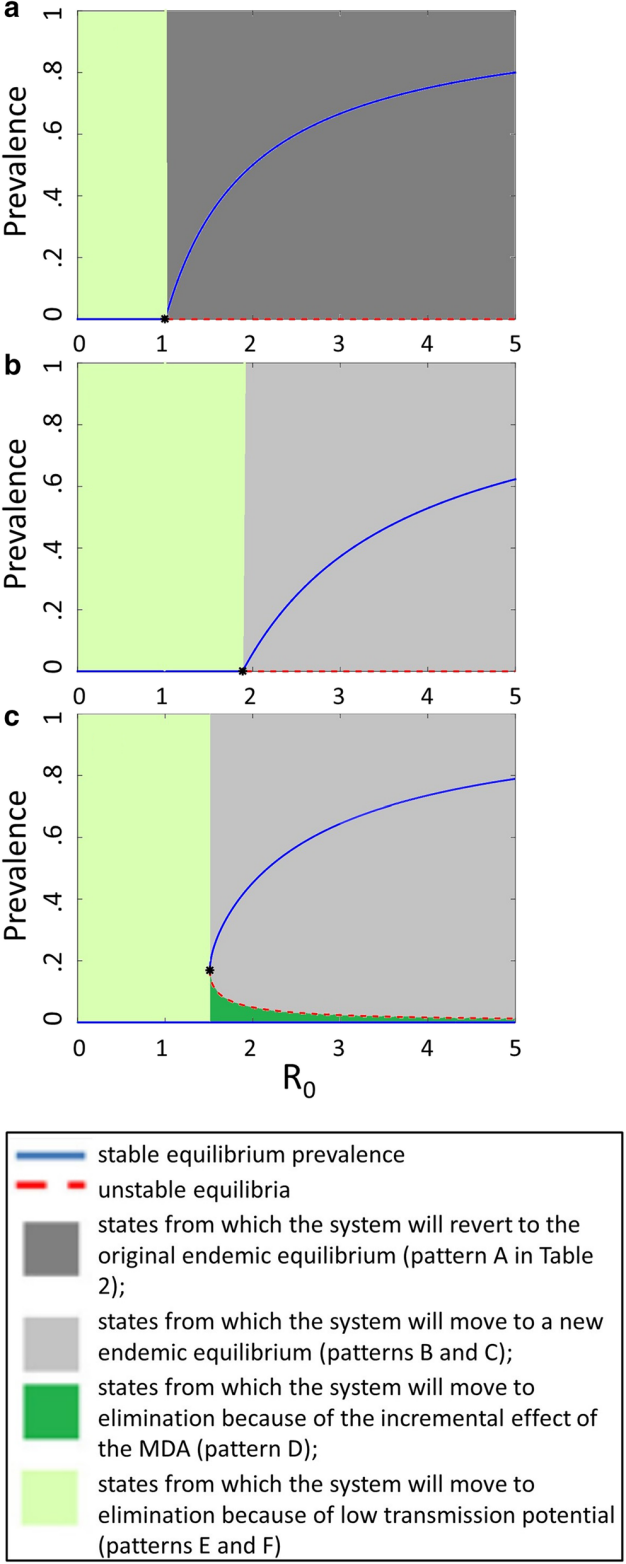
Schematics of bifurcation diagrams for different scenarios. **a** Gives the general patterns expected when there is no intervention; at any non-zero value of the prevalence it will move vertically towards the blue line *R*_0_. **b** Scenario with reduced transmission potential. **c** Scenario with a small reduction in transmission potential and a saddle-node bifurcation

The trends in Fig. 1 were then used to develop a classification of the potential outcomes of programmes. This analysis of trends is complemented by bifurcation diagrams [16] showing how the equilibrium prevalence achieved by the programme depends on *R*_0_ (Fig. 2). These diagrams are intended only to illustrate possible qualitative patterns. Both Figs. 1 and 2 are supported by qualitative arguments advanced in a previous publication [5, 17] and by the results of mathematical models published elsewhere [17–20].

### Microsimulation model

Micro-simulations were used to obtain approximate quantitative results applicable for more or less realistic transmission settings, taking into account factors of secondary importance such as the duration of chemoprevention and levels of acquired immunity. Using *OpenMalaria* (https://github.com/SwissTPH/OpenMalaria/wiki), a single site individual-based simulation model of malaria in humans [21] linked to deterministic model of malaria in mosquitoes [22]. The simulations track parasite densities, corresponding to different infection events, with different sub-models for infection of humans [23], and for blood-stage parasite densities with the main immune effects controlling parasite densities and infection duration [24]. Clinical malaria, and hence routine case management and passive case detection, is triggered by hyper-parasitaemia, with the models for both parasite densities and clinical incidence calibrating using the data of various field studies [24, 25]. *OpenMalaria* include various sources of variation between hosts in exposure and susceptibility, while treating the infections in mosquitoes as perfectly mixing.

### Simulation of interventions

The simulations considered effects of MDA, of routine case management (treatment of clinical malaria attacks) and of the package of additional interventions (test-and-treat). MDA was simulated via a single round of DHA-P treatment, parameterized as described previously [26]. This implementation captures both the effects of truncating infections, and also the subsequent prophylactic effect based on pharmaco-kinetic/dynamic studies. The efficacy of the programme is defined as the relative percentage drop in average parasite prevalence in 2 to 10 year olds from the 3 years pre-intervention to the average value 7–10 years post-MDA. Routine case management was simulated in *OpenMalaria* as described previously [13, 27]. The set of *OpenMalaria* simulations of test-and-treat were parameterized by simulating a test-and-treat strategy, where a positive from a rapid diagnostic test (with a diagnostic cut-off of 50 parasites per μl of blood and a specificity of 0.942, based on fitting to the data of Murray et al. [28] results in cure of blood-stage parasites at the next time-step.

The simulated test-and-treat programme was additional to the routine case management system, and comprised treatment of a fixed number ($\nu$) of the neighbours of passively-detected index cases. The number of index cases investigated each week ($\upiota$) was set either to a pre-defined maximum ($\upiota_{\text{max} }$), or to the number of cases presenting for treatment during 1 week, whichever was greatest. To simulate targeting, the number of simulated individuals to be tested in the same interval was inflated by the targeting index ($\tau$), equivalent to the ratio of the prevalence in all individuals tested (including asymptomatics) to that in the general population [17], which was set to a constant value within a simulation. The application of this approach to account for spatial clustering and heterogeneity in OpenMalaria is described in detail elsewhere [29]. The effective per capita rate of testing was then: $\Phi = \frac{\iota \nu \tau }{N}$ per week, where $N$ is the population size. Treatment rates implemented in *OpenMalaria* were rescaled to allow for the 5-day simulation time-step.

### Simulation experiment

For analysis of resurgence rates simulations were carried out using the *OpenMalaria* R0000 base model parameterization [30] with a simulated population size of 10,000 (other exploratory simulations that used the R0670 parameterization, which allows for heterogeneity in susceptibility to co-morbidity, gave the same general results). The same pattern of seasonality of transmission based on the pattern for southern Zambia used in previous analyses [13] was used throughout. This was scaled to achieve a pre-determined annual entomological inoculation rate during the initial phase of the simulation.

The pre-existing intervention programme was assumed to include health-facility based routine case management with a defined effective coverage, *E*_14_, (per 14 day period) [31] to have been in place for long enough for an approximate stable endemic state to have been reached by the time the test-and-treat programme is introduced. Values of *E*_14_ of 16% (corresponding to a weak health care system) and 84% (corresponding to a very strong health care system) were simulated.

It is assumed that in any real situation it would be impracticable to introduce both MDA and test-and-treat programmes at the same time, and it would be illogical to commence a test-and-treat programme after the MDA was completed, since resurgence might then occur before the programme that might avert it was in place. The MDA was, therefore, carried out 4 years after the test-and-treat programme was initiated.

The importation rate is relevant because the intervention package needs to be able to prevent onward transmission from imported cases. To test sensitivity to importation, simulations were carried out with a value similar to a previously published estimate for Zanzibar [32].

To examine how the transmission level, the effective coverage of case management, the importation rate, and the parameters of the test-and-treat programme affect the outcome, a factorially designed simulation experiment was carried out with each combination of the levels listed in Table 1 for each factor. Each such scenario was simulated with each of 3 different seeds, both with and without one round of MDA at 90% coverage, leading to a total of 16,200 population simulations. These simulations were performed at the sciCORE (http://scicore.unibas.ch/) scientific computing core facility at University of Basel.

**Table 1:** Summary of factor levels in simulation experiment

Variable	Units	Levels simulated
Initial EIR	Infectious bites per person per annum	3, 4, 5, 6, 8
Effective coverage of case management (E_14_)	Proportion of clinical malaria cases receiving effective treatment within a 14 day period	16%, 84%
Importation rate	Imported infections per 1000 population per year	0, 1.6
Maximum number of index cases ($\upiota_{\text{max} }$)	Maximum number of index cases investigated per week	1, 2, 3, 4, 5
Targeting ratio ($\uptau$)	Ratio of the size of a random sample that would be need to be tested and treated, to the number actually treated, in order to achieve the same number of effective treatments	1, 1.5, 2, 2.5, 3, 3.5, 4, 4.5, 5
Neighbours tested $\left( \upnu \right)$ per index case	number of neighbours of passively-detected index cases investigated	5, 10, 15

### Analysis of elimination and resurgence rate

Point prevalence for the whole population was extracted at an interval of 30 days (simulation time) throughout each simulation. Each simulation with MDA was then matched with the corresponding non-MDA simulation, and each pair of simulations was classified according to whether:

i. There was a significant prevalence reduction induced by the test-and-treat programme alone. This was evaluated using a paired t-test to compare prevalence in the year before introduction, with that 11 years later at the same time of year. A 5% significance level was used to define statistical significance.
ii. Elimination occurred. This was defined as an average prevalence less than a threshold (set to 0.005 except where indicated otherwise). Sensitivity analyses were used to evaluate the effect on the classification of alternative thresholds. A criterion of zero infections would be biased because some simulations included importation and would therefore have non-zero prevalence even if local transmission was minimal.

The resurgence rate was measured by analysis of the rate of approach of the time-point specific prevalence in the simulation with MDA, $p_{I} \left( t \right)$, to that of controls without MDA (matched on all other aspects of the scenario), $p_{C} \left( t \right)$. Justified by the patterns observed in simulations with resurgence, the resurgence process was approximated with an exponential function of the form:

1 $$\frac{{p_{C} \left( t \right) - p_{I} \left( t \right)}}{{p_{C} \left( t \right) - p_{\min}}} = \exp\left( { - \lambda t} \right),$$

where $p_{\min} = p_{I} \left( 0 \right)$ denotes the minimum prevalence achieved in the year after MDA, $\lambda$ is the resurgence rate, and $\left( {ln2} \right)/\lambda$ is the half-life of resurgence, and where time-dependent values $p_{C} \left( t \right)$ are used as comparators with $p_{I} \left( t \right)$ in order to allow for the seasonal forcing.

Values for the 4 years after this minimum were analysed by fitting a linear regression model to the logarithmic transform of Eq. (1), in order to estimate $\lambda$ separately for each matched pair of simulations. Resurgence was said to occur in simulations where there was no elimination and for which the half-life was less than 10 years. Where elimination did not occur, the long-term percentage reduction in prevalence was computed from the ratio of average prevalence during years 9–11 after the round of MDA, to that in the year before the test-and-treat began.

## Results

### Conceptual model

The analysis in the present study concerns settings where routine control measures, such as vector control and case-management, have been scaled up maximally but where *P. falciparum* malaria remains endemic. The situation initially approximates an endemic stable state, with minimal fluctuations in yearly prevalence and incidence. The intervention programme is then enhanced by adding components of test-and-treat, MDA or both.

Figure 1a illustrates schematically the time course of average transmission potential in such a population for each strategy as quantified by the controlled reproduction number, *R*_*c*_. In the absence of either test-and-treat or MDA, *R*_*c*_ remains constant (the yellow line in Fig. 1a). Prevalence also remains constant (yellow lines in Fig. 1b, c). MDA and test-and-treat both reduce *R*_*c*_. MDA leads to a transient dip in *R*_*c*_ as a result of the prophylactic effect of the intervention (blue line in Fig. 1a). In addition, by clearing parasitaemia, a single round of MDA has an immediate, but transient effect on the infectious reservoir, leading to more extended effects on prevalence. Even if transmission is interrupted by the MDA, in the absence of effective surveillance reintroductions will occur and both *R*_*c*_ and prevalence will return to the initial stable states (blue lines in Fig. 1a–c) [5, 9].

The test-and-treat programme is envisaged as targeted investigations around passively-detected cases of existing infections, with resources for these investigations remaining constant over time, once the programme is initiated. The test-and-treat programme is assumed to be added to the routine case management system, which serves as a passive surveillance component of a surveillance-response system. It functions by testing and treating neighbours of passively-detected index cases. In Fig. 1a, the introduction of test-and-treat is represented by a very rapid decrease in *R*_*c*_ (the red line). In response to the reduced value of *R*_*c*_, prevalence decreases gradually, either until a new endemic stable state is reached (red line in Fig. 1b), or until transmission is interrupted (Fig. 1c).

While there is substantial empirical and theoretical support for the broad patterns shown for both MDA and test-and-treat in Fig. 1, it is not obvious what may be achieved by combining a pulse of MDA with scale-up of test-and-treat. The black lines in Fig. 1b, c illustrate schematically hypothetical trajectories for the prevalence when the two interventions are combined, with the test-and-treat introduced shortly before a pulse of MDA. Table 2 lists possible results of adding MDA to a test-and-treat programme (as assessed in terms of the time-course of prevalence), with patterns C and E corresponding to the dashed lines in Fig. 1b, c, respectively, and patterns D and F, corresponding to the solid black lines in Fig. 1b, c, representing the desired outcomes. Patterns A and B correspond to cases where the final prevalence remains similar when MDA is added, to that achieved with test-and-treat alone.

**Table 2:** Patterns resulting from combining one round of MDA with sustained test-and-treat programmes

Pattern	Effect of test-and-treat alone	Incremental effect of MDA	Resurgence	Number of OpenMalaria simulations (%)
A	Minimal change to stable state	Transient effect only	Yes^a^	2124 (26.2%)
B	New endemic stable state	Transient effect only	Yes	4953 (61.1%)
C	New endemic stable state	Speeds up achievement of lower stable state	No	522 (6.4%)
D	New endemic stable state	Elimination	No^b^	246 (3.0%)
E	Elimination	Minimal speed up in achievement of elimination	Yes	62 (0.8%)
F	Elimination	Speed up in achievement of elimination	No	193 (2.4%)

^a^48 of these simulations were classified as no-resurgence, because the effect of MDA was minimal

^b^59 of these simulations were classified as resurgent, because there was a brief prevalence increase after MDA, followed by a subsequent crash classified as interruption of transmission

**Fig. 3:**
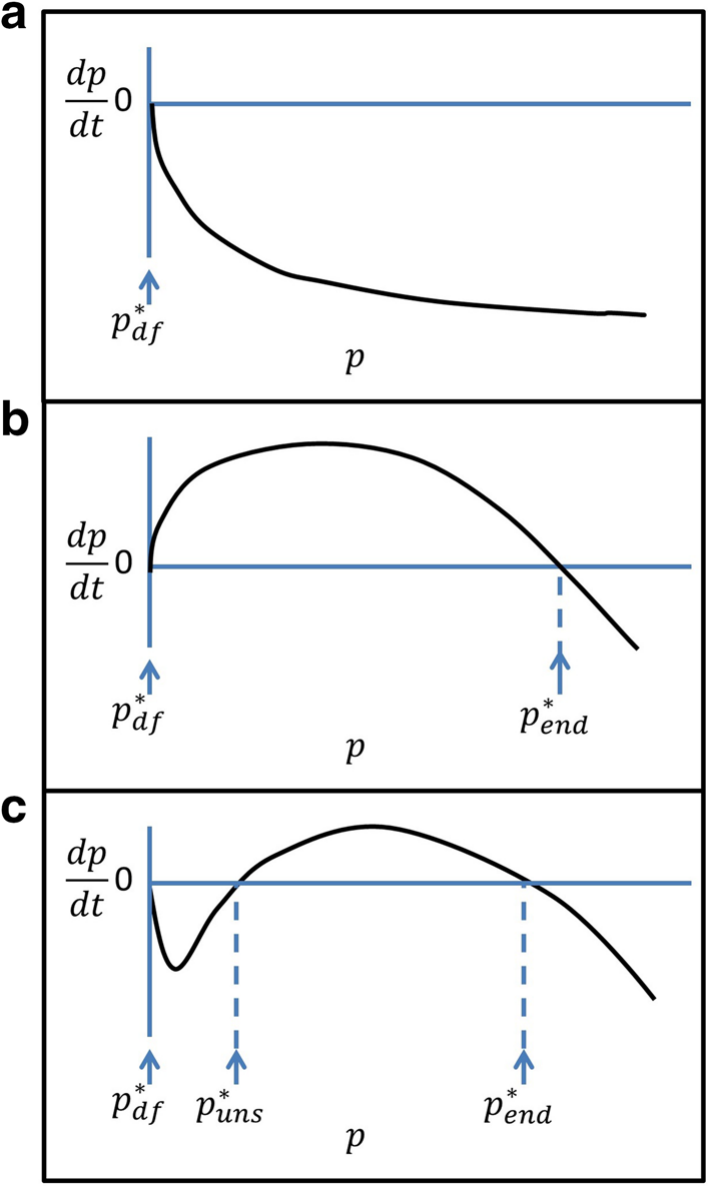
Schematics of equilibria at different values of *R*_*c*_. **a** *R*_*c*_ less than the bifurcation, $R_{c}^{*}$ (occurs in Fig. 2a–c); **b** $R_{c} > R_{c}^{*}$, without bistability (occurs in Fig. 2a, b); **c** $R_{c} > R_{c}^{*}$, with bistability (occurs in Fig. 2c). $p_{df}^{*}$ is the disease-free equilibrium which is stable in **a** and **c**, and unstable in **b**; $p_{uns}^{*}$ is an unstable equilibrium; $p_{end}^{*}$ is an endemic stable equilibrium

Equivalent to the question of whether MDA will be followed by resurgence is that of what will be the subsequent trajectory of prevalence if a system at equilibrium is disturbed by removing a substantial proportion of the infections. Figure 2 (which is derived from a very simple (susceptible-infected-susceptible; SIS) model of malaria dynamics in humans [17]) illustrates how the possible outcomes listed in Table 2 arise as functions of the minimum prevalence achieved after the round of MDA, and of the dynamics induced by the intervention programme. Figure 3 indicates how such systems adjust to perturbations at each prevalence value with each panel corresponds to different verticals in Fig. 2, and the vertical axis giving the rate of change (arbitrary units) over time in the prevalence, following a perturbation to the equilibrium; $dp/dt = 0$, corresponds to an equilibrium.

In systems conforming to the pattern in Fig. 2a the prevalence will resurge to the stable equilibrium (the blue line) (see also Fig. 3a, b). The only exceptions are (i) if there is independently a change in the transmission potential, $R_{\text{c}}$, so that the prevalence now falls into the left-hand part of the diagram, where the disease-free equilibrium is stable, or (ii) if infections have been cleared entirely, and there are no reintroductions (in which case the system remains at the unstable, disease-free equilibrium, corresponding to the red dashed line). If interventions are introduced that reduce the reproduction number to some new value $R_{\text{c}}$, (corresponding to a lower endemic level) they may change the diagram into one like Fig. 2b. Qualitatively the system has not changed, but there is an extended range of settings over which elimination is occurs (corresponding to outcome E or F in Table 2).

Alternatively, the system may exhibit bi-stability (Fig. 2c). This occurs when there is a range of $R_{\text{c}}$ values for which there are two stable (blue line) prevalences, separated by a non-zero unstable equilibrium at some intermediate prevalence, $p_{uns}^{*}$. This range always has a lower bound but does not necessarily have an upper bound [17]. Bistability arises if infectiousness or persistence of infections is lower at lower prevalence and is an inherent characteristic of some mathematical models of immunity, in particular, those that include hosts that are temporarily resistant [16, 18] or with exposure-dependent clearance rates [20]. In such systems, once elimination is achieved it can be maintained even in the face of low levels of introduction, providing that $p_{uns}^{*}$ is not exceeded [19, 20]. In some such systems the bifurcation occurs in the absence of interventions, and at a value of $R_{0} < 1 \left[ {16,18} \right]$. However simple malaria models without interventions (such as Ross-Macdonald, or the even simpler SIS model obtained by reducing the mosquito model to a constant factor [17]), generally give diagrams like Fig. 2a, where there is no bistability, implying that clearing parasites (e.g. with MDA) in an endemic setting, will at best lead to an unstable parasite-free state, where any reintroductions will lead to resurgence.

The endemic equilibrium in OpenMalaria (and in the microsimulation model of Griffin et al. [33]), increases monotonically with $R_{0}$ and this has been interpreted as implying that there is no bistability [34]. However, while such non-monotonicity does lead to bistability, it is not a necessary condition (as illustrated by Fig. 2c). Moreover, there may be a qualitative change in the bifurcation diagram from one of the topology of Fig. 2b to that of Fig. 2c depending on the interventions that are in place [17].

### Results of microsimulations

**Fig. 4:**
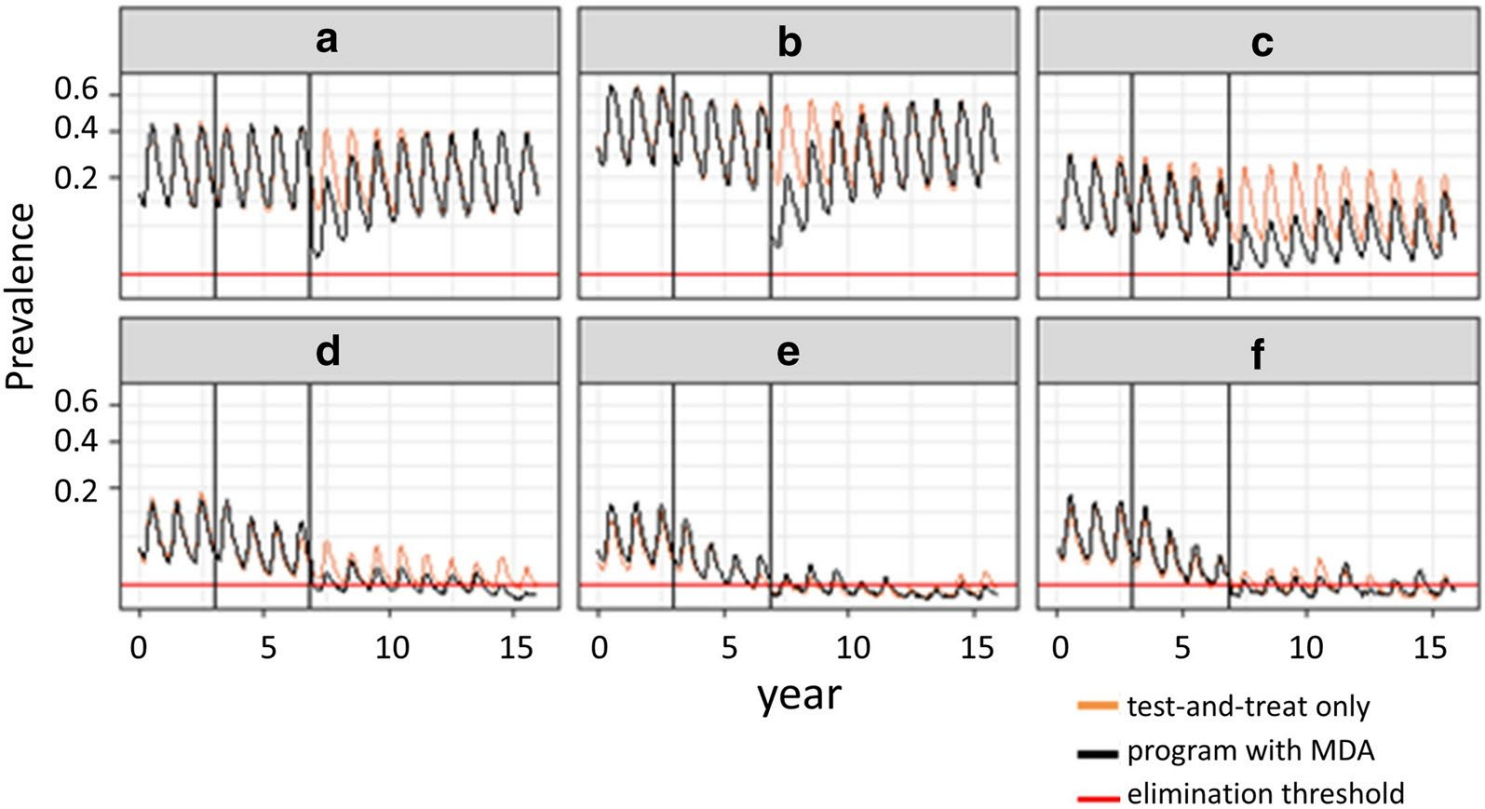
Time profiles of prevalence for typical simulations. Simulated prevalence over time for archetypical scenarios exemplifying the six patterns described in Table 2. There is no active intervention in the first 3 years, then test-and-treat is begun and maintained from then on. MDA is carried out 4 years after the test-and-treat begins. The vertical lines correspond to the times when programme reorientation occurs

Typical time profiles of prevalence in the microsimulations are shown in Fig. 4.

These were analysed to determine the minimum prevalence achieved, the average prevalence during the resurgent period, and the resurgence rates, enabling each pair of simulations to be classified according to which of the patterns listed in Table 2 they match. Each simulation could be classified into one of the six patterns, and examples of simulated time-series of prevalence corresponding to each pattern are given in Fig. 4. Most fell into patterns A–C where there was no elimination. In 3.2% (255) of the 8100 matched pairs of simulations there was elimination in the control (patterns E and F), but only 3.0% (246) exhibited pattern D, where the MDA made a difference to whether elimination was achieved (Table 2). Even when there was elimination defined using the parasite density cut-off, the simulated EIR (during the 4 years after the MDA) was non-zero (Table 3), though at values of less than 0.1 infectious bites per person-year. These EIR levels are clearly too low to sustain transmission and would be unmeasurable in the field, but do lead to small numbers of secondary cases, which can explain the seasonal oscillations in prevalence in simulations classified as having achieved elimination.

**Table 3:** Simulated transmission and treatment rates during the 4 years after MDA (medians and inter-quartile ranges)

CM coverage (E14)	Pattern	Entomological inoculation rate (EIR, infectious bites per person-year)		Rate of routine case management (treatments per 1000 person-years)		Screening rate (tests per 1000 person-years)		Ratio of facility treatment rate to EIR		Ratio of community treatment rate to EIR	
		Simulations with MDA	Control simulations	Simulations with MDA	Control simulations	Simulations with MDA	Control simulations	Simulations with MDA	Control simulations	Simulations with MDA	Control simulations
84%	A	1.39 (0.82, 2.43)	2.71 (1.89, 4.04)	67.2 (50.3, 82.5)	99.2 (87.8, 105.9)	38.1 (26.5, 52.3)	39.9 (27.4, 53.6)	48.4 (33.9, 61.3)	36.2 (26.1, 46.8)	3.6 (2.5, 4.9)	3.1 (2.2, 4.3)
84%	B	0.56 (0.28, 1.03)	1.26 (0.67, 2.00)	36.5 (21.6, 54.1)	66.3 (44.7, 83.8)	116.9 (79.9, 171.4)	129.3 (88.0, 194.0)	65.3 (52.5, 78.3)	52.1 (40.9, 66.4)	11.9 (8.2, 17.5)	11.4 (7.6, 17.8)
84%	C	0.12 (0.07, 0.19)	0.47 (0.32, 0.73)	10.4 (6.7, 15.5)	36.8 (25.9, 49.3)	86.5 (49.8, 135.1)	111.7 (67.0, 181.3)	87.9 (80.7, 99.0)	75.3 (66.7, 86.9)	10 (5.7, 15.3)	11.3 (6.7, 18.2)
84%	D	0.04 (0.02, 0.06)	0.22 (0.14, 0.32)	3.6 (1.9, 5.7)	19.9 (13.5, 28.7)	63.2 (31.0, 101.5)	103.1 (64.9, 161.7)	99.5 (91.3, 107.8)	90.5 (85.1, 96.8)	7.2 (3.6, 11.4)	10.7 (6.8, 16.2)
84%	E	0.04 (0.03, 0.06)	0.07 (0.04, 0.14)	4.1 (3.0, 5.8)	7.2 (3.7, 12.8)	117.9 (90.7, 179.0)	144.6 (106.5, 204)	101.7 (91.9, 107.9)	97.2 (89.5, 102.4)	12.1 (9.6, 18.3)	15.2 (11.4, 19.9)
84%	F	0.02 (0, 0.03)	0.08 (0.04, 0.13)	1.7 (0.3, 3.3)	7.9 (4.1, 12.3)	58.8 (15.9, 109.6)	131.9 (96.8, 191.0)	97.9 (81.4, 107.8)	94.8 (89.2, 99.6)	6.7 (2.1, 11.7)	13.3 (10.1, 18.1)
16%	A	1.55 (0.92, 2.28)	2.79 (1.91, 3.78)	11.4 (9.2, 13.0)	15.2 (14.1, 15.9)	36.5 (25.1, 47.0)	39.1 (27.1, 51)	7.3 (5.7, 10.1)	5.5 (4.2, 7.5)	5.7 (3.8, 7.5)	5.1 (3.5, 6.8)
16%	B	0.87 (0.43, 1.51)	1.71 (0.99, 2.57)	8 (5.5, 10.6)	12.2 (9.9, 14.0)	106.8 (77.5, 152.6)	121.4 (87.6, 179.5)	9.2 (6.9, 12.3)	7.2 (5.3, 9.9)	17.9 (13, 25.6)	17.4 (12.0, 26.4)
16%	C	0.14 (0.1, 0.18)	0.58 (0.4, 0.76)	2.1 (1.4, 2.9)	7.2 (5.5, 8.9)	83.2 (62.3, 100.3)	139.7 (103.0, 177)	16.2 (14.9, 17.6)	12.6 (12.0, 13.4)	19.7 (15.5, 23.7)	27.9 (20.1, 35.2)

In total 88.3% (7150 of 8100) of simulations with MDA were classified as having resurgence with a median half-life of resurgence of 2.3 years (inter-quartile range 1.1–2.8). When elimination was not achieved, the MDA had minimal effect on the percentage reduction in final prevalence (median reduction 0.7%, inter-quartile range − 2%, 5%).

**Fig. 5:**
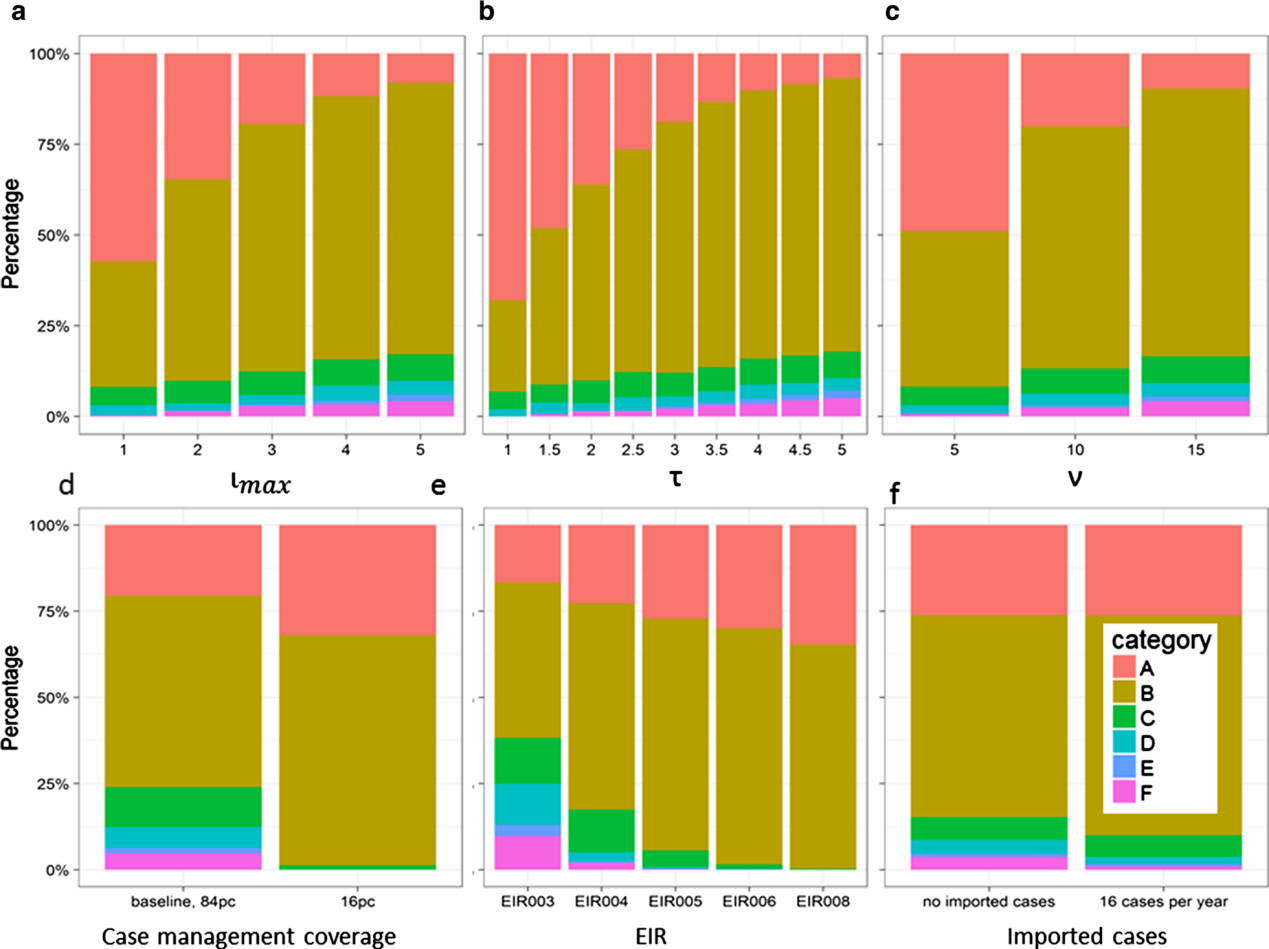
Percentages of simulations by outcome pattern. Percentages of simulations classified according to the six categories described in Table 2. The different panels correspond to the variables listed in the first column of Table 1

Because of the factorial design, the marginal averages are unbiased estimates of the effects of the individual factors that varied by design. The parameters of the test-and-treat programme rarely affected whether elimination occurred (Fig. 5a–c), but had a large effect on the proportion of simulation pairs exhibiting patterns A or B, indicating that intensive test-and-treat changes the steady-state prevalence, when sustained over a long period (in this case of approximately a decade). Even in the simulations without MDA there was a substantial reduction in the EIR (from the median value of 5 infectious bites per person-year), resulting from the effect of the test-and-treat (Table 3).

**Fig. 6:**
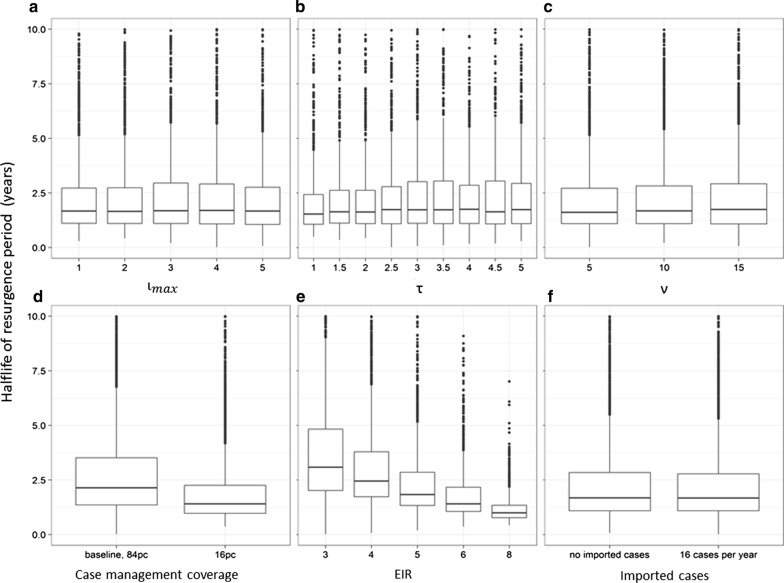
Distributions of resurgence rates. Half-life of resurgence by factors listed in Table 1. Boxes correspond to the median, and the 25th and 75th percentiles (the hinges). Whiskers extend from the hinges to the most extreme values within 1.5 times the Inter Quartile Range. Outliers are shown as points. Only scenarios with resurgence (as defined in “Methods”) are included. The different panels correspond to the variables listed in the first column of Table 1

The greater the intensity of test-and-treat, the greater the long-term effect on the prevalence (as assessed by the approximate steady state in years 9–11 after MDA). At the same time, the parameters of the test-and-treat programme had little effect on the resurgence rate in those simulations where there was resurgence (Fig. 6a–c).

Case management coverage remained the same throughout the simulation (it was not scaled-up, or introduced part-way through the programme, like the test-and-treat intervention), so its consequences result from interactions with the other interventions.

These included a very dramatic effect on the proportion of simulations leading to elimination (Fig. 5d). Elimination occurred in none of the simulations at the low case management coverage. At the same time, only 23% (832) of non-eliminating MDA simulations with high case management coverage had pattern A (where the final state was not significantly different from the pre-MDA one) compared with, 32% (1292) of the corresponding simulations with low case management coverage, indicating that the test-and-treat strategy is more likely to lead to an accumulated effect on prevalence if routine case management is at high coverage. Related to this, the resurgence rate was lower in those simulations with high case management coverage (Fig. 6d).

**Fig. 7:**
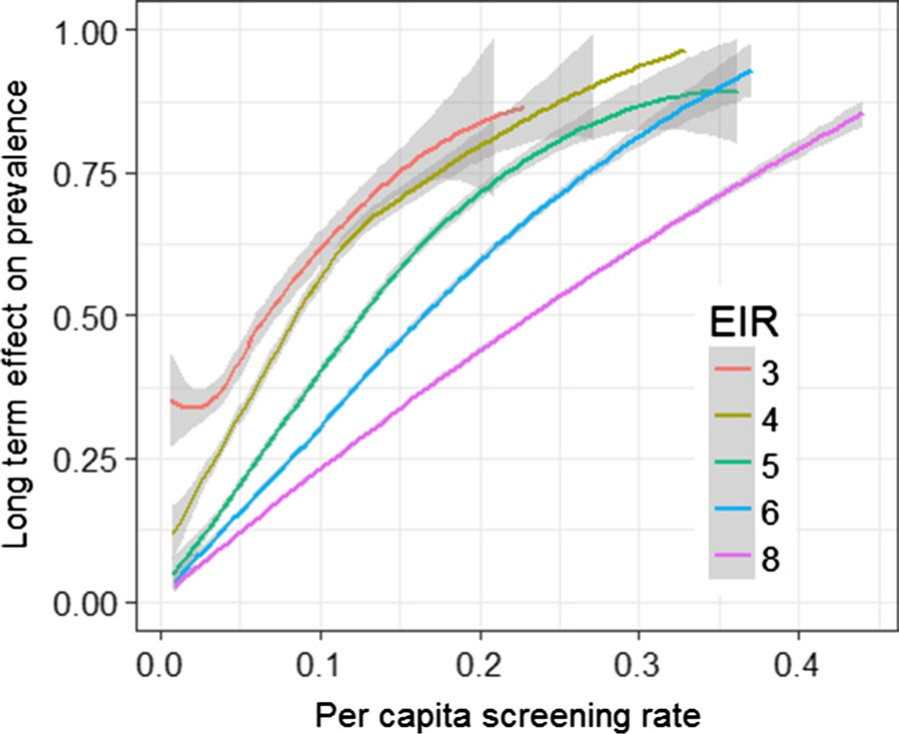
Effect of combined programme on prevalence (non-elimination scenarios). Long-term reduction in prevalence computed as the reduction in the ratio of average prevalence during years 9–11 after the round of MDA, to that in the year before test-and-treat began. The EIR value is that applicable before the introduction of the programme. The grey envelopes correspond to 95% confidence intervals around Loess-smoothed lines

The transmission potential, as measured by the initial EIR, also had a dramatic effect on the percentage of simulations leading to elimination (Fig. 5e). 15% (485) of simulations of initial EIR ≤ 4 exhibited elimination, but in only 16/4860 (0.3%) of simulations with higher initial EIR was there elimination. Twelve of these exhibited pattern D (elimination in the control), and only 4 had pattern E or pattern F, indicating that the MDA had minimal effect on the long-term outcome if the EIR exceeded this rather modest value. In the simulations where elimination occurred with MDA, the control simulations were ones with very low control EIR during the post-MDA period (Table 3). When elimination did not occur, the long-term effect of the test-and-treat programme on prevalence was greater in low (initial) EIR simulations (Fig. 7) and in these simulations the resurgence rate was slower than with a higher EIR (Fig. 6e).

Imported infections decreased the proportion of simulations leading to elimination (Fig. 5f), but did not completely avert elimination when case management coverage was high. Importation, however made no difference to the final steady state in the non-elimination scenarios, and hence did not affect the proportion of simulations with pattern A, or the rate of resurgence when there was no elimination (Fig. 6f).

It is impracticable to draw bifurcation diagrams for the OpenMalaria model, because it is both computationally expensive and stochastic, but the conceptual model (Fig. 2) can account for the occurrence of the different outcome patterns (Fig. 5) in terms of the impact of the interventions on transmission potential, and whether they induce bistability. Where transmission is interrupted in simulations with MDA, but not without MDA this implies that the system is bistable. Since this occurs only in simulations with high case-management coverage, it implies that high case management coverage can induce bistability.

Where the treatment rate (considering both treatment of clinical cases and of infections diagnosed via test-and-treat strategies), increases less than proportionately with prevalence, the system is bi-stable. A simple theoretical analysis (Additional file 1) indicates why this arises when the effective coverage of case management is high, even when there is no bistability in the model without case management. The same analysis indicates that test-and-treat (as simulated here) reduces transmission by greater proportion at higher levels of prevalence, and so does not induce bistability.

In the simulations, this differential effect of high coverage of case management, compared with the test-and-treat programme is reinforced by the relationship between simulated treatment rates and the EIR during the period after MDA (Table 3).

**Fig. 8:**
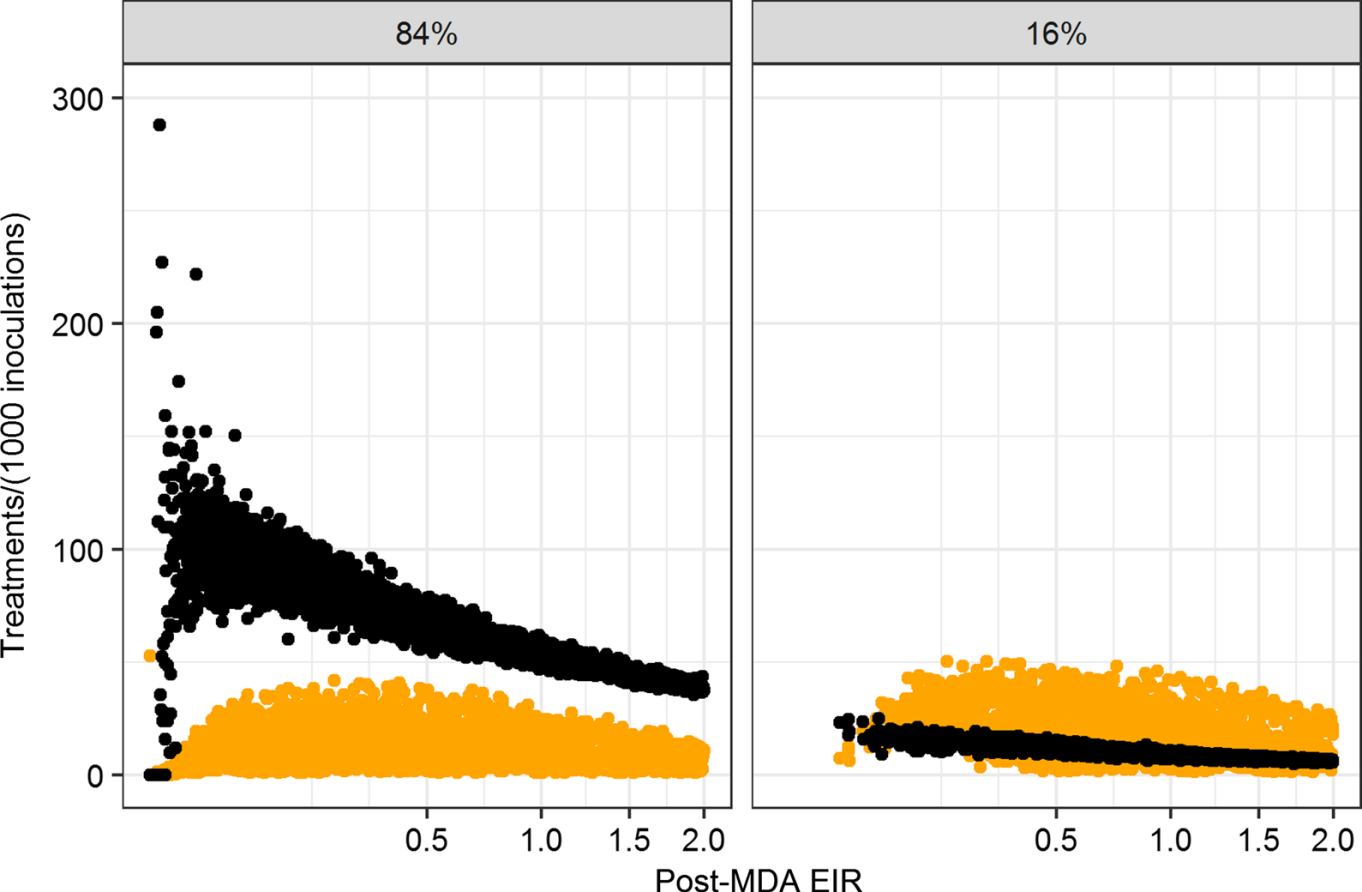
Ratio of treatments to residual EIR. Each point corresponds to the ratio of the mean number of treatments during the 4 years after MDA, to the mean EIR during the same period, for one simulation; filled black circle: treatment of clinical cases; filled orange circle: treatment via the test-and-treat programme. The different panels correspond to the different coverages of case management. The EIR axis is on a square-root scale to emphasize the behaviour at very low EIR

Following MDA there were substantial declines in both the EIR and the rates of case management below the levels in the control simulations. This was found in all outcome groups. However, the decrease in in the numbers of treatments of clinical cases was less than that in the EIR so the ratio increased in the scenarios where transmission was reduced to the lowest levels (Fig. 8a), generally reaching the highest levels in the elimination scenarios with patterns D–F (Table 3) (though at the very lowest levels of residual EIR there was considerable stochasticity in the number of treatments). At low case management coverage (Fig. 8b), the residual EIR never fell into the range where this ratio was substantial.

The number of simulated treatments resulting from the test-and-treat programme did not adjust in the same way to the residual EIR. At very low EIR, (and hence low case-incidence) the effectiveness of the test-and-treat is limited by the number of index cases, i.e. $\iota < \iota_{\text{max}}$. At higher residual EIR the effective screening rate $\Phi = \Phi_{\text{max} } = \frac{{\iota_{\text{max} } \nu \tau }}{N}$ is constant within any given scenario, and is independent of transmission, so the consequent rate of treatment is proportional to the prevalence (Table 3, Fig. 8) (and hence the ratio to the EIR decreases somewhat with increasing EIR, owing to saturation).

## Discussion

Field trials of malaria elimination strategies including multiple interventions are challenging undertakings, and it is impossible to trial all the different strategies that might be considered. In silico trials using microsimulation models such as *OpenMalaria* make it possible to test a wide range of different strategies, calibrated to approximate real settings, so that the analyses allow in a quantitative way for complicating factors such as seasonality, acquired immunity, and case importation. In the simulations reported here, MDA led to elimination in only a small proportion of the simulations, and in most scenarios there was resurgence, despite the very high coverage of the intervention. Test-and-treat strategies also only contributed to elimination in only a small number of the simulated scenarios.

Concepts derived from simpler models, such as the Ross-Macdonald [35] model, or even SIS compartment models that reduce the complexity of the system to a minimum [17] are invaluable for understanding why these rather disappointing results were obtained, and for deducing under which circumstances success might be achieved.

The impact of MDA was simulated as equivalent to that of a single round of MDA with high (90%) coverage, a rather optimistic coverage level. In principle, MDA programmes can consist of any number of rounds, each potentially covering different overlapping subsets of the population. However, previous *OpenMalaria* simulations suggested that the impact can mostly be explained by the escape probability, defined as the proportion of the population that never receive any MDA [26]. Other factors compromising coverage (such as seasonal migration [36]) could also be captured by effect on the escape probability. A low escape probability (10%) thus errs on the side of the optimistic, while approximating many different patterns of coverage of multiple MDA rounds.

The small proportion of the scenarios in which elimination occurred is readily explained on the basis of the inferred form of the bifurcation diagram. MDA is a pulsed intervention with only a transient effect on $R_{\text{c}}$ (which arises because of the prophylactic effect and the truncation of existing infections). Only in a subset of settings is there bi-stability and only in a subset of these does MDA move the system abruptly below $p_{uns}^{ *}$ and into the dark green zone in Fig. 2c and thus achieve elimination. This is consistent with simple stochastic models for the probability of extinction by MDA, which indicate that this is extremely unlikely unless the population size is very small and the coverage of MDA extremely high [37].

The existence of the bi-stability that enables elimination depends crucially on the coverage of case management. In various models of clinical malaria, the ratio of the incidence of clinical attacks to the number of inoculations is higher in lower prevalence settings. If there is significant treatment in reaction to these attacks, this leads to the pattern of bifurcation diagram of Fig. 2c, with a saddle-node bifurcation at the lowest value of $R_{\text{c}}$ at which transmission persists. In such systems, elimination following MDA may be a stable state, even if it is not achieved without MDA. This can arise if the health system functions better when malaria is near-absent or when prevalence is very low [19] or if new infections are more likely to be treated in low prevalence settings where there is less clinical immunity [19]. With *OpenMalaria* this pattern is induced by highly effective case management as a result of the selection effect described in Additional file 1, which requires neither of these preconditions. At values of $R_{\text{c}}$ higher than that at the saddle-node there are two possible non-zero equilibrium prevalence values (Fig. 2c). The lower branch of unstable endemic equilibrium points divides the basins of attraction of the disease-free equilibrium point and the larger endemic equilibrium points. If the prevalence is greater than the unstable endemic equilibrium point, then the prevalence will resurge until it reaches the larger endemic equilibrium point. If the prevalence after MDA is less than this unstable equilibrium point, $p_{uns}^{*}$, then the infection dies out and case management will lead to elimination.

At the simulated importation rate and coverages of case management, imported infections did not affect whether resurgence occurred, indicating that the case management alone was sufficient to control importations in those cases where MDA had brought prevalence below $p_{uns}^{*}$. If prevalence is reduced to zero, then importation is necessary for the system to escape from an unstable disease-free equilibrium, but this is a highly unlikely scenario since the disease-free equilibrium is only achieved when there is a high coverage of case management. Since importation has minimal effects on resurgence rates, it is unlikely to be an important determinant of outcomes in such settings.

In contrast, the simulations reported here agree that the transmission potential is both the primary determinant of whether elimination can be achieved by a malaria intervention programme, and also of the rate of resurgence after MDA when it cannot. Because of its modest impact on $R_{c}$, the coverage of case management has only a small effect on the resurgence rate, despite its critical role in determining whether resurgence occurs at all. If coverage of case management is low, reducing transmission leads to diagrams like that of Fig. 2b, where the threshold value of $R_{0}$ (where the endemic equilibrium meets the axis) is increased, but where there is no bistability. With this form of bifurcation diagram, MDA will not increase the range of settings where elimination occurs.

Test-and-treat strategies do not lead to bi-stability in the scenarios shown in this paper, and only contribute to elimination via their modest reduction in average transmission. Test-and-treat accelerates the approach to elimination only in a subset of those simulations where the test-and-treat programme already rapidly reduces prevalence. About the same number of simulations were classified into patterns F (elimination with no resurgence, arguably equivalent to acceleration) and E (elimination following temporary resurgence), but in all these cases, the prevalence in both control and MDA simulations was already very low by the time of the MDA (Fig. 4).

Untargeted test-and-treat is an inefficient way of reducing average transmission because its coverage is linearly related to the reproduction number (in contrast to killing adult female mosquitoes which is related via an exponential function of coverage [38]), so it is unsurprising that test-and-treat leads to elimination in only a small subset of simulations. In principle, effective targeting can substantially improve the impact of test-and-treat. This may also lead to bistability if the targeting ratio, $\uptau$, decreases with prevalence [17]. The test-and-treat simulated here in *OpenMalaria* treats $\tau$ as fixed (and also assumes that the resources allocated to the test-and-treat system, measured by the product of $\iota$ and $\nu$, are constant). These are likely to be reasonable approximations to recurrent focal screen-and-treat or recurrent focal MDA, especially where targeting in space and time is rather imprecise, but give a poor representation of highly targeted reactive case detection. There is a need to evaluate microsimulations of reactive case detection approaches where $\uptau$ may adapt over time [17].

## Conclusions

The main determinants of whether resurgence will occur following MDA are the residual transmission potential and the routine treatment of passively detected cases. A high coverage of case management is essential for elimination, but in most simulated scenarios, transmission was not interrupted and the transmission potential proved the main determinant of the resurgence rate. Other reactive interventions, such as high intensity reactive case detection and reactive vector control might also be effective in averting resurgence at low transmission potential and require further analysis.

## Supplementary information

**Additional file 1.** Differential effects of case management and test-and-treat on infectiousness.

## Data Availability

The underlying code and simulation results are available on request from the authors.

## Abbreviation

MDA: Mass Drug Administration
