# Supplementary material for: Resurgence of malaria infection after mass treatment: a simulation study
Source: Malar J. 2019 Dec 5;18:409. doi: 10.1186/s12936-019-3019-0 (PMC6896478; doi:10.1186/s12936-019-3019-0)
Supplement: Supplementary file 1 — Additional file 1. Differential effects of case management and test-and-treat on infectiousness. [file 12936_2019_3019_MOESM1_ESM.docx]

**Differential effects of case management and test-and-treat on infectiousness**

#### Additional Material to:

#### Resurgence of malaria infection after mass treatment: a simulation study

Thomas A Smith, Peter Pemberton-Ross, Melissa Penny, Nakul Chitnis

Define *κ* as the infectiousness of the human population over some arbitrary time-interval, (possibly measured by the proportion of mosquitoes that become infected at each feed). This can be analysed as two components:

$\kappa=\lambda w_{1}\kappa_{1}+pw_{2}\kappa_{2}$

where the first component, $\lambda w_{1}\kappa_{1}$, is the contribution of new infections arising during the interval (proportional to $\lambda$, the force of infection) and $pw_{2}\kappa_{2}$ is the contribution of the standing crop of pre-existing infections (proportional to $p$, the prevalence).$\kappa_{1}$ and $\kappa_{2}$ thus represent the different average infectiousness of the different categories of infection while $w_{1}$ and $w_{2}$ are constant scale-factors.

Clinical malaria disproportionately occurs during the initial period of an infection. Treatment of some proportion, $\varepsilon_{1}$, of clinical malaria cases thus reduces the first component, and hence the overall infectiousness to:

$\kappa_{CM}=\lambda{\left( 1-\varepsilon_{1} \right)w}_{1}\kappa_{1}+pw_{2}\kappa_{2}$

and the proportion by which the overall infectiousness is reduced is:

$1-\frac{\kappa_{CM}}{\kappa}=\frac{\lambda{\varepsilon_{1}w}_{1}\kappa_{1}}{\lambda w_{1}\kappa_{1}+pw_{2}\kappa_{2}}$

which is a decreasing function of *p.*

Conversely, test-and-treat applied to some proportion, $\varepsilon_{2}$, of the infected population reduces the second component, and hence the overall infectiousness to:

$\kappa_{TT}=\lambda w_{1}\kappa_{1}+\left( 1-\varepsilon_{2} \right)pw_{2}\kappa_{2}$

and the proportion by which the overall infectiousness is reduced to:

$1-\frac{\kappa_{TT}}{\kappa}=\frac{p{\varepsilon_{2}w}_{2}\kappa_{2}}{\lambda w_{1}\kappa_{1}+pw_{2}\kappa_{2}}$

which is an increasing function of *p.*

This analysis assumes that the proportions, $\varepsilon_{1}$ and $\varepsilon_{2}$ are independent of prevalence.
